# Investigating microcrystalline cellulose crystallinity using Raman spectroscopy

**DOI:** 10.1007/s10570-021-04093-1

**Published:** 2021-07-27

**Authors:** Ana Luiza P. Queiroz, Brian M. Kerins, Jayprakash Yadav, Fatma Farag, Waleed Faisal, Mary Ellen Crowley, Simon E. Lawrence, Humphrey A. Moynihan, Anne-Marie Healy, Sonja Vucen, Abina M. Crean

**Affiliations:** 1 SSPC Pharmaceutical Research Centre, School of Pharmacy, University College Cork, Cork, Ireland; 2 SSPC Pharmaceutical Research Centre, School of Chemistry, University College Cork, Cork, Ireland; 3 SSPC Pharmaceutical Research Centre, School of Pharmacy, Trinity College Dublin, Dublin, Ireland

**Keywords:** Microcrystalline cellulose, Raman spectroscopy, Crystallinity, Partial least square regression, R Shiny

## Abstract

Microcrystalline cellulose (MCC) is a semi-crystalline material with inherent variable crystallinity due to raw material source and variable manufacturing conditions. MCC crystallinity variability can result in downstream process variability. The aim of this study was to develop models to determine MCC crystallinity index (%CI) from Raman spectra of 30 commercial batches using Raman probes with spot sizes of 100 µm (MR probe) and 6 mm (PhAT probe). A principal component analysis model separated Raman spectra of the same samples captured using the different probes. The %CI was determined using a previously reported univariate model based on the ratio of the peaks at 380 and 1096 cm^−1^. The univariate model was adjusted for each probe. The %CI was also predicted from spectral data from each probe using partial least squares regression models (where Raman spectra and univariate %CI were the dependent and independent variables, respectively). Both models showed adequate predictive power. For these models a general reference amorphous spectrum was proposed for each instrument. The development of the PLS model substantially reduced the analysis time as it eliminates the need for spectral deconvolution. A web application containing all the models was developed.

**Graphic abstract:**

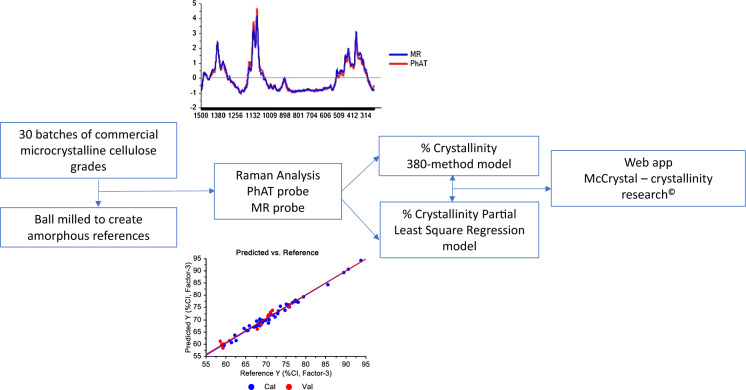

**Supplementary Information:**

The online version contains supplementary material available at 10.1007/s10570-021-04093-1.

## Introduction

Microcrystalline cellulose is widely used and has diverse applications across different industry sectors. MCC is used as a binder and filler in pharmaceutical formulations, a fat replacement and stabilizer in food products, a rheology control agent in cosmetics, and as a component of biodegradable polymers and wooden products (Gibis et al. 2015; Terinte et al. 2011; Thoorens et al. 2014; Vonbehren et al. 2010; Yang et al. 2018). The most common source of MCC is wood. Cellulose chains are present in wood pulp in the form of packed layers that are held together by lignin, and strong hydrogen bonds (Thoorens et al. 2014). MCC is obtained by purification of wood using mineral acid solution, i.e. acid hydrolysis, followed by rinsing and drying. The presence of hydroxyl groups in the product of this purification process and the relatively large surface to volume ratio of micro fibrils give rise to MCC’s hygroscopic character (Sun 2008).

MCC has an atypical semi-crystalline structure and its attributes can vary between suppliers and batches. Batch to batch variability can be caused by different factors, such as wood source (hard or soft wood), climate differences from region to region, harvesting time, the process of pulp delignification, hydrolysis reaction time, and the process of drying (O’Regan 2018; Rowe et al. 1994). The term crystallinity index (%CI) refers to the percentage by weight occupied by the crystallites (Foster et al. 2018). During depolymerization (hydrolysis) the acid preferentially attacks the amorphous regions of the pulp (Landín et al. 1993).

MCC crystallinity has been reported to influence its behaviour during processing. Tabletability was investigated with differences observed between batches with substantial differences in crystallinity. Suzuki and Nakagami used a rod mill to reduce crystallinity of a MCC batch from an initial %CI of 65%. A reduction in tabletability was observed for batches with a %CI below 12%, and an increase in dissolution rate was observed for acetaminophen tablets produced with MCC that had a %CI less than 26% (Suzuki and Nakagami 1999). The crystallinity of MCC has also been shown to influence water sorption (Amidon and Houghton 1995; Bolhuis and Chowhan 1996; Nokhodchi 2005). Increased water sorption was observed with decreased MCC crystallinity, as moisture sorption occurs predominately in amorphous regions which are more hydrophilic than the crystalline regions (Mihranyan et al. 2004; Segal et al. 1959; Suzuki and Nakagami 1999).

A range of techniques has been utilised to determine MCC crystallinity. Diffraction techniques are most widely reported, employing the Segal peak height method (Segal et al. 1959), peak decomposition or deconvolution (Lanson 1997; Park et al. 2010; Ahvenainen et al. 2016; Yao et al. 2020), and Rietveld refinement based methods (Madsen et al. 2011; Ling et al. 2019). Other spectroscopic techniques proposed include Fourier transform infrared (Liu and Kim 2015), solid state NMR (Atalla and Vanderhart 1984; Harris et al. 2012; Wickholm et al. 1998), and Sum frequency generation (Ling et al. 2019). Raman spectroscopy, the focus of this study, has been investigated to determine cellulose crystallinity (Agrawal et al. 2010; Agarwal et al. 2018; Agarwal 2019). An initial Raman approach for quantifying MCC crystallinity employed relatively weak bands at 1462 and 1481 cm^−1^ (CH_2_ bending modes) in conjunction with spectral deconvolution (Schenzel et al. 2005). Two further methods were proposed employing bands at 380 and 93 cm^−1^ (Agarwal et al. 2018, 2010). The 93 cm^−1^ method is advantageous compared to the 380 cm^−1^ method as it differentiates crystalline and organized cellulose and an aggregated form which is not crystalline. However, the 93 cm^−1^ method requires an FT-Raman instrument with 1064 nm excitation to avoid Rayleigh scattering that masks the sample Raman scattering at 93 cm^−1^.

The quantification of cellulose crystallinity using Raman spectroscopic analysis has primarily employed instruments with laser spot sizes between 50 μm and 1 mm and limited depth of penetration (Agarwal et al. 2018, 2010; Foster et al. 2018). The irradiation area of such instrumental setups results in a limited area being sampled. Therefore, analysis requires the acquisition of multiple spectra at a number of locations to obtain a representative profile of the sample. Reduced depth of penetration also results in spectra that focus on surface spectral features. To date Raman probes with larger laser spot sizes and depth penetration, designed for non-contact analysis of solids, have not been applied to the quantification of cellulose crystallinity. The larger sample volume irradiated using these probes would reduce the requirement for multiple spectra acquisition and surface mapping.

The objective of this study was to demonstrate the capability of Raman spectra acquired using non-contact Raman probes to predict the crystallinity index of commercial MCC batches. The %CI in this study refers to the ratio between the amount of crystalline cellulose and the total amount of sample. Raman spectra acquired for 30 commercial MCC batches, using two probes with spot sizes of 100 µm (MR probe) and 6 mm (PhAT probe), were used to develop models to determine %CI.

## Materials

Thirty batches of commercial microcrystalline cellulose were studied (Table S1 supplemental data). Samples comprised MCC manufactured by 3 different suppliers; 25 batches from Dupont Nutrition & Biosciences, 4 batches from JRS Pharma, and 1 batch from Asahi Kasei Corporation. All MCC samples analysed compiled with USP/NF, Ph.Eur and JP pharmacopeia. Samples analysed encompassed a range of different MCC grades which varied in average particle size distribution, bulk density and moisture content; PH101, PH102, PH200, 90 M. Wood pulp was confirmed as the botanical source for 29 of the 30 batches analysed, for one batch the botanical source could not be confirmed whether it came for a cotton or wood source.

## Methods

### Milling standards to produce reference amorphous samples

Milling was performed in order to obtain amorphous reference spectra. Prior to milling, the samples were kept in the oven at 40 °C for 24 h. To produce amorphous reference materials for each batch 1 g of MCC was milled at 25 Hz in an oscillatory ball mill, Mixer Mill MM400 (Retsch GmbH, Germany), in order to decrease the crystallinity (Mattonai et al. 2018). All samples were milled for 90 min to replicate the methodology of previous studies (Agarwal et al. 2010). A break of 15 min was performed after every 30 min of milling operation. Samples were confirmed to be amorphous after 90 min milling by powder X-Ray diffraction (PXRD). A representative PXRD diffractogram of a batch before and after ball milling is included Fig. S2 in the supplemental material.

### Powder X-ray diffraction

Powder X-Ray diffraction (PXRD) analysis was performed using a Stoe Stadi MP diffractometer operating in transmission mode, with a tube voltage of 40 kV and current of 40 mA, using Cu Kα1 monochromated radiation (1.5406 Å) and a gas-filled PSD detector. MCC powder samples were held between acetate foils and the diffractogram was collected between 10° and 30° 2θ. Environmental background was removed by subtracting a blank diffractogram (2 acetate films with no sample) from all spectra.

### Preparation of MCC pellets for Raman spectroscopy

Cylindrical, flat, 13 mm diameter, 250 mg pellets were produced using an Atlas 15 T Manual Hydraulic Press (Specac Ltd, Orpington, UK). Three tonnes were applied for a duration of 30 s. Two pellets were produced for each batch; one from the powder ‘as received’ and one from the corresponding ball milled sample.

### Raman spectroscopy

Raman spectra for each pellet were acquired using two different probes. The first was a MR probe connected to a RamanRxn™ instrument (Kaiser Optical Systems Inc., Ann Arbor, USA), with nominal laser beam diameter at a focal position of 100 μm. The exposure time set was 60 s, using a laser power of 785 mW, over the range 200–1500 cm^−1^, and analysis was performed in triplicate. The second was a PhAT probe connected to a RamanRxn_2_PhAT™ instrument (Kaiser Optical Systems Inc., Ann Arbor, USA), with nominal laser beam diameter at a focal position of 6 mm. The exposure time was set to 15 s, using a laser power of 785 mW, over the range 200–1500 cm^−1^, and analysis was performed in duplicate. Moreover, while the MR probe is primarily a surface technique, the PhAT probe has a collection zone depth of around 2 mm.

In addition to the sample Raman fingerprint, the spectra obtained contained a background contribution that may be caused by fluorescence or thermal fluctuations on the Charge Coupled Device (CCD detector) (Bocklitz et al. 2011; Gautam et al. 2015). The fluorescence background was removed by pre-processing. The spectra underwent a baseline subtraction of an interpolated linear fit between the anchor points fixed on the X axis: 1500, 1200, 952, 857, 743, 632, 550, 260, and 200 cm^−1^. The intensity differences observed between the spectra were removed by standard normal variate (SNV). This normalization consisted of subtracting each spectrum from the mean and dividing the result by the spectrum standard deviation.

### Univariate determination of crystallinity index

Crystallinity index was calculated according to the method proposed by Agarwal et al., which is based on the ratio between the intensity of the peaks at 380 cm^−1^ and 1096 cm^−1^ deconvoluted from a reference amorphous spectrum (referred to henceforth as the “380-method”) (Eq. 1) (Agarwal et al. 2010). Deconvolution in this study refer to the extraction of the amorphous and the crystalline spectral contributions from the actual measured spectrum. Deconvolution is required because cellulose Raman spectra are composed of the amorphous and crystalline spectra superimposed (Agarwal et al. 2010). The ratio of these peaks was compared to other peaks ratios and showed efficiency and great sensitivity to cellulose crystallinity changes (Agarwal et al. 2018, 2010).

1 $$\% CI = \frac{{\left( {{\raise0.7ex\hbox{${I_{380} - I_{380\_am} }$} \!\mathord{\left/ {\vphantom {{I_{380} - I_{380\_am} } {I_{1096} - I_{1096\_am} }}}\right.\kern-\nulldelimiterspace} \!\lower0.7ex\hbox{${I_{1096} - I_{1096\_am} }$}}} \right) - 0.0286}}{0.0065}$$

I_380_ and I_380_am_ are the intensities at the Raman shift 380 cm^−1^ of the commercial batch as received and its amorphous corresponding sample, respectively. I_1096_ and I_1096_am_ are the intensities at the Raman shift 1096 cm^−1^ of the commercial batch as received and its amorphous corresponding sample, respectively.

The spectra of corresponding amorphous samples were obtained by ball milling a sample of the batch, pressing the powder into a pellet, and acquiring spectra of the pellet. Each Raman spectrum was then pre-processed and peak normalized by equalizing the intensity values at Raman shifts above 857 cm^−1^ to the intensity at 857 cm^−1^, following the method previously reported by Agarwal et al. (Agarwal et al. 2010). The resulting spectrum was considered to represent the amorphous contribution to the Raman spectra of the sample as received (Agarwal et al. 2010). Thus, in Eq. 1, I_380_am_ and I_1096_am_ are equal to the intensity of the peaks at 380 cm^−1^ and 857 cm^−1^ in the spectrum of the milled sample, respectively.

### Correction of 380-method due to Raman instrument-dependence

The 380-method was originally developed using a Raman instrument RFS-100 (Bruker Inc.) and the coefficients in Eq. 1 (used to calculate %CI) are specific to the study’s instrumental setup (Agarwal et al. 2010). Equation 1 was established by plotting the intensity ratios of interest from the Raman spectra against %CI of a calibration set samples determined using a PXRD peak intensity methodology (Segal et al. 1959). It is important to note that the %CI values determined by the 380-method show instrument-dependence and therefore it is recommended that a calibration be performed should the instrumental set-up alter (Foster et al. 2018). Therefore. It was necessary to perform a calibration study to correct this methodology for the specific Raman instruments used in the this study, i.e. the MR and the PhAT probes connected to a RamanRxn™ and a RamanRxn_2_PhAT™ (Kaiser Optical Systems Inc., Ann Arbor, USA), respectively.

A single MCC batch (batch #7) was selected to create a series of MCC samples with a range of crystallinity values for the calibration study. To create samples with varying crystallinity, binary mixtures MCC prior to ball milling and MCC after ball milling (amorphous) were prepared at a total mass of 0.20 ± 0.03 g. The blends prepared contained 100%, 83%, 72%, 58%, 44%, 33%, and 22% w/w MCC prior to ball milling. The crystallinity index (${\%CI}_{PXRD}$) of each blend was determined from its PXRD diffractogram (Eq. 2)

2 $$\% CI_{PXRD} = \frac{{\left( {I_{200} - I_{am} } \right)}}{{I_{200} }}$$

*I*_200_ is the averaged intensity in the range 22.55–22.65° 2θ representing the crystalline peak, and *I*_*18.7*_ is the averaged intensity in the range 18.0.65–18.75° 2θ corresponding to the amorphous scatter (Segal et al. 1959). PXRD diffractograms for individual blends are shown in Fig S2 (supplemental data).

$\% CI_{PXRD}$ determined for each blend from the PXRD data was plotted against the peak ratio of the Raman wavenumbers 380/1096 cm^−1^ after amorphous contribution subtraction. The amorphous contribution subtraction was described in "Raman spectroscopy" section. A linear regression between the theoretical %CI and the ratio 380/1096 cm^−1^ was obtained for each instrument and the linear equation obtained was used to determine the %CI by 380-method ("Raman spectroscopy" section) for all other batches.

### Principal component analysis

Principal component analysis (PCA) was performed in order to identify differences between the Raman spectra obtained for the 30 commercial batches of MCC investigated. Unscrambler® X 11.0 software (CAMO software, Norway) was used to perform the analysis on the pre-treated (baseline and SNV) Raman spectra. The algorithm NIPALS and cross validation were performed with 29 segments determined so that spectra of the same batch acquired using both probes were kept within the same segment to avoid overfitting. A total of 145 spectra were used to build the PCA model.

### Partial least square regression

Two partial least square regression models were built using Unscrambler® X 11.0 software (CAMO software, Norway) aiming to predict crystallinity index from Raman spectral analysis of the 30 commercial MCC batches. One model was built using the pre-treated (baseline and SNV) Raman spectra acquired using the MR probe and another model using the pre-treated spectra acquired using the PhAT probe. The intensity of the pre-treated Raman spectra between 1500 and 250 cm^−1^ were used as X variables. For both models, the Y variable was the %CI determined by the 380-method. The algorithm Kernel was used. A total of 24 batches were used to calibrate the model and 6 batches were used to validate the model. The number of spectra used were 85 and 60 for MR probe and PhAT probe models, respectively.

### Shiny web application

A web application, McCrystal–crystallinity research^**©**^**,** was built using ‘shiny’ (Chang et al. 2019) version 1.4.0 in R (R Core Team 2019) using the development environment RStudio (RStudio Team 2019). This application was built to facilitate the dissemination of the models developed in this study. The web application framework for R was developed in tab set panels using the package ‘shinydashboard’ (Chang and Borges Ribeiro 2018) version 0.7.1. The package ‘RcppArmadillo’ (Eddelbuettel and Sanderson 2014) version 0.9.800.3.0 was used to manipulate matrices, graphics were built using the package ‘ggplot2’ (Wickham 2016) version 3.2.1, the spectra were normalized using the package ‘prospectr’ (Stevens and Ramirez-Lopez 2013) 0.1.3, and manipulated using the package ‘spectrolab’ (Meireles et al. 2018) version 0.0.8. Spectra baseline, i.e. linear interpolation between predetermined points, was performed using the package ‘spftir’ (Pozo Valenzuela and Rodriguez-Llamazares 2016) version 0.1.0 and ‘pracma’ (Borchers 2019) version 2.2.9. After the PCA analysis, samples were clustered in 2-D scores plots using the package ‘cluster’ (Maechler et al. 2019) version 2.1.0. The package ‘basicTrendline’ (Mei and Yu 2018) version 2.0.3 was used to plot the trendline between scores and crystallinity index. This web application can be accessed at https://sspc.ie/mccrystal-registration/. The design code is available in the supplementary material.

## Results

### Raman spectra fluorescence background removal

**Fig. 1:**
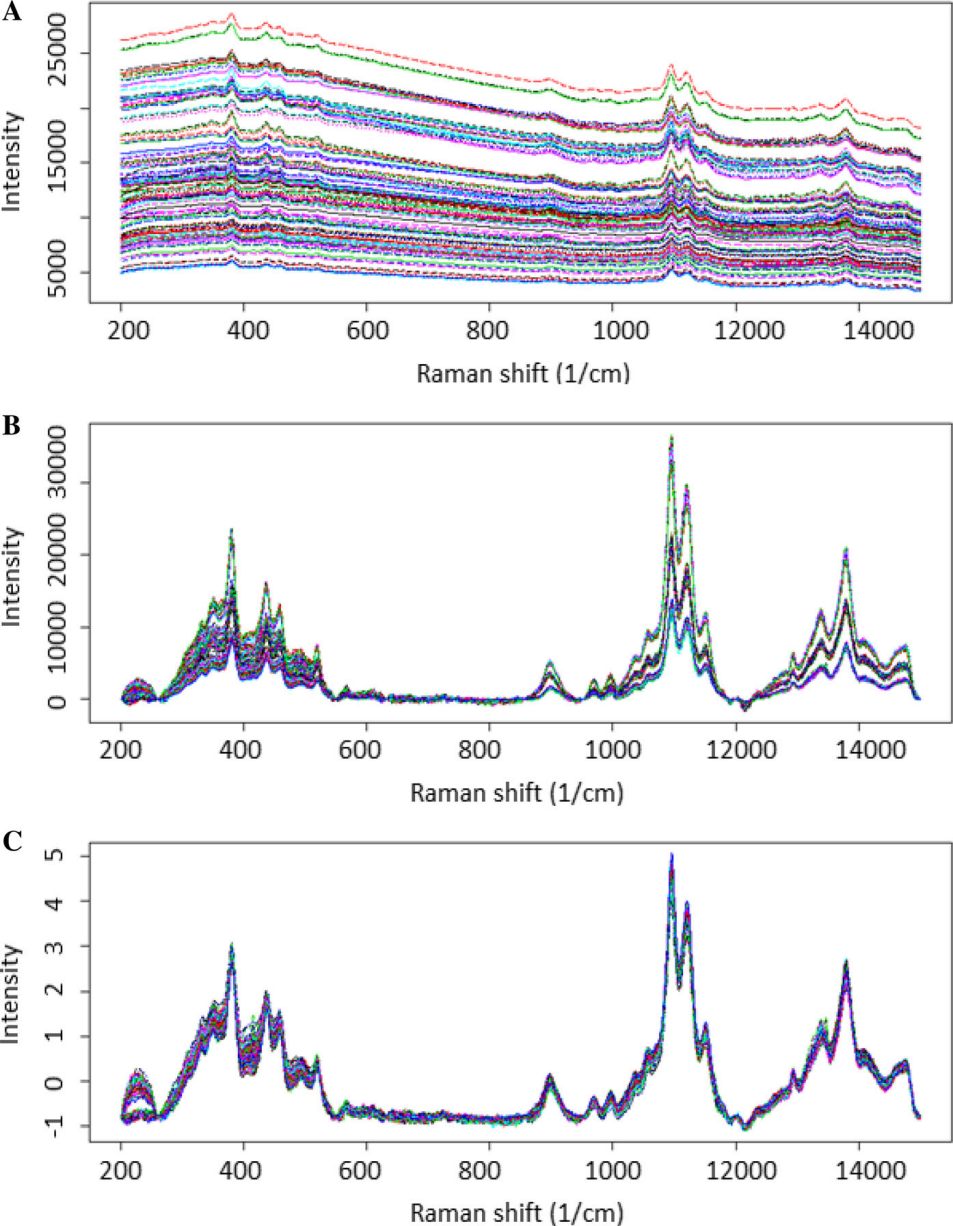
Spectra acquired by PhAT probe **a** raw spectra exhibiting the fluorescence background and intensity shifts, **b** spectra following baseline transformation, and **c** spectra following SNV and baseline transformation

Prior to establishing the crystallinity models from Raman spectra, it was necessary to remove spectral interference. Fluorescence background and intensity shifts were observed (Fig. 1). The baseline subtraction method developed ("Raman spectroscopy" section) eliminated the fluorescence background and standard normal variate (SNV) eliminated intensity differences observed between the spectra. The SNV normalization consisted of subtracting each spectrum by its mean and dividing the result by the spectrum standard deviation. All spectra used in this study were baselined and SNV normalized accordingly.

### Principal component analysis of spectra

**Fig. 2:**
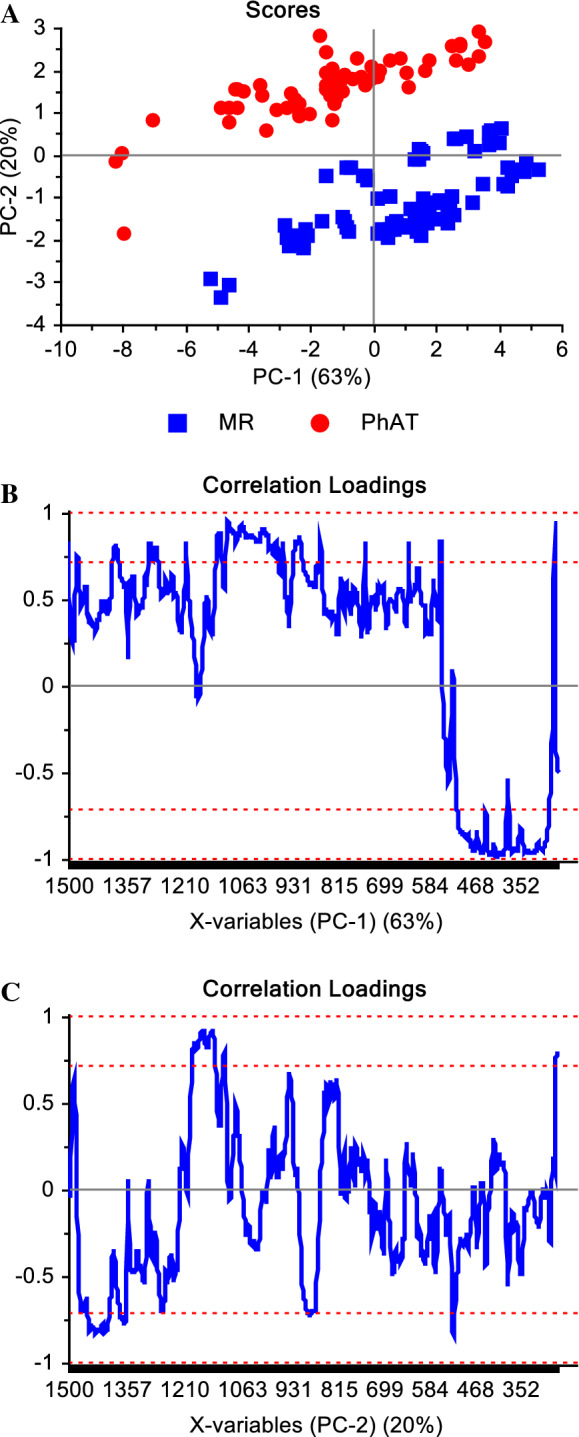
**a** PCA Scores plot, **b** correlation loadings of the first principal component and **c** correlation loadings of the second principal component of the model built using spectra of 30 MCC batches acquired with MR (n = 85 spectra) and PhAT (n = 60 spectra) probes

**Fig. 3:**
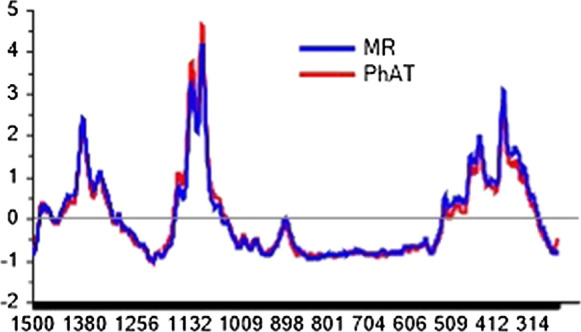
Spectra acquired using a MR and a PhAT probe after baseline correction and SNV normalization for a sample of single batch

A PCA model was used to explore spectral differences due to the probe set-up used. For this purpose, the model was built from treated spectra (baseline and SNV) acquired using both, MR and PhAT probes. The scores plot (Fig. 2a) show a clear separation of the samples into two groups based on the spectra acquired using either the MR probe or the PhAT probe. The Raman shifts that lie within the upper and lower bounds of the correlation loadings plot are the regions where the variability was modelled by that principal component (Fig. 2b and 2c). Variability in the regions containing the peaks at 380 and 1096 cm^−1^ are captured by the PCA model. Spectral comparisons showed that the peak intensity at 1096 cm^−1^ is higher and at 380 cm^−1^ is lower for the PhAT probe in comparison to the MR probe. Figure 3 provides representative spectra for a single batch highlighting that differences in intensities were observed for spectra acquired using the different probes, and these differences could not be removed by baseline correction or SNV. This is a strong indication that the crystallinity index determined using spectral data from a MR probe cannot be compared to the crystallinity index determined using spectra data from a PhAT probe.

### Development of specific 380-method equations for MR and PhAT Raman instruments

**Fig. 4:**
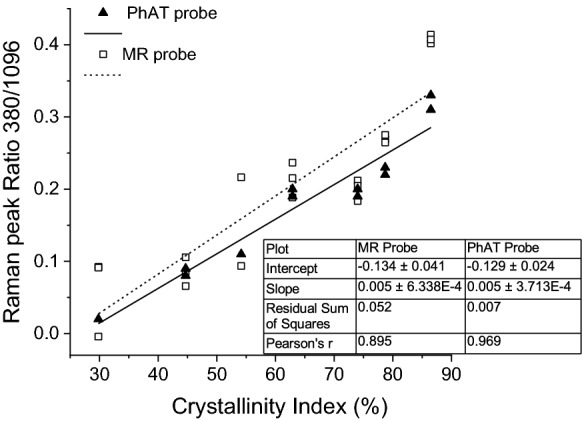
Correlation between the ratio of the Raman intensities at 380 and 1096 cm^-1^, and the crystallinity index determined for PXRD data using the Segal method (Segal et al. 1959). Pre-treated deconvoluted spectra obtained for blends using MR and PhAT probes were used

Linear regression plots of %CI_PXRD_ determined from PXRD diffractograms for the calibration sample set ("Correction of 380-method due to Raman instrument-dependence" section) and the ratio between the Raman intensities at 380 and 1096 cm^−1^ showed correlation for spectra acquired using both the MR and PhAT probes. Pearson’s r values of 0.895 and 0.969 for the MR probe and PhAT probe, respectively were obtained (Fig. 4). Differences in the linear relationship can be explained by the capacity of each probe to capture the intrinsic crystallinity heterogeneity of MCC samples. A PhAT probe averages a larger area (12.57 mm^2^) in comparison to a MR probe (7.85 × 10^–3^ mm^2^). Thus, the PhAT probe was able to capture a more representative measurement of the sample. This may explain why the PhAT probe showed a better correlation to the PXRD crystallinity index, %CI_T_. Replicate spectra acquired by the PhAT probe also provided more consistent peak ratio values for the same sample. Figure 4 contains replicates (n = 3 for MR probe and n = 2 for PhAT probe) and it was clear that the replicate PhAT probe measurement deviated less than the MR probe measurements.

Based on the correlations of Fig. 4, specific 380-method equations were proposed for MR and PhAT instrumental systems, Eqs. 3–4, respectively.

3 $$\% CI_{MRprobe} = \frac{{\left( {{\raise0.7ex\hbox{${I_{380} - I_{380\_amorphous} }$} \!\mathord{\left/ {\vphantom {{I_{380} - I_{380\_amorphous} } {I_{1096} - I_{1096\_amorphous} }}}\right.\kern-\nulldelimiterspace} \!\lower0.7ex\hbox{${I_{1096} - I_{1096\_amorphous} }$}}} \right) + 0.134}}{0.005}$$

4 $$\% CI_{PhATprobe} = \frac{{\left( {{\raise0.7ex\hbox{${I_{380} - I_{380\_amorphous} }$} \!\mathord{\left/ {\vphantom {{I_{380} - I_{380\_amorphous} } {I_{1096} - I_{1096\_amorphous} }}}\right.\kern-\nulldelimiterspace} \!\lower0.7ex\hbox{${I_{1096} - I_{1096\_amorphous} }$}}} \right) + 0.129}}{0.005}$$

### Determination of MCC crystallinity indexes for commercial batches

**Fig. 5:**
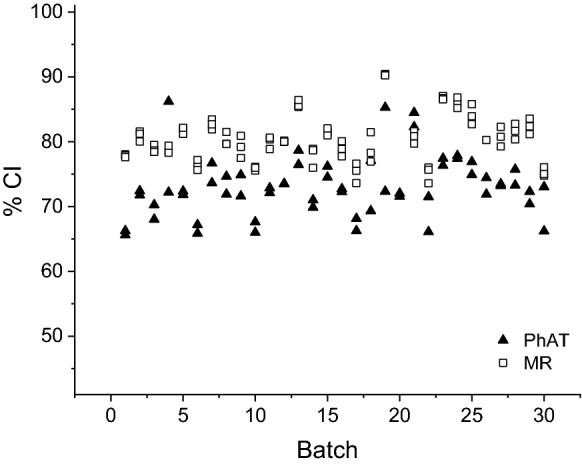
Crystallinity index (%CI) determined for commercial batches using spectra obtained for pellets and different probes (MR probe n = 3, and PhAT probe n = 2, using Eq. 3 and 4, respectively). Individual %CI values reported are included in supplemental material, Table S1

The %CI for a set of 30 commercial batches was determined using the corrected 380-method (Eqs. 3 and 4) applied to spectral data acquired using the MR and PhAT probes, respectively. Amorphous spectra were obtained for each MCC batch with both probes. From the processed amorphous spectra from each batch obtained using the MR probe (n = 70) and PhAT probe (n = 55), an averaged amorphous spectrum was generate for both probes. The averaged intensities and standard deviations for the peaks of interest for the MR probe averaged amorphous spectrum were I_380_am_ (0.627 ± 0.246) and I_1096_am_ (2.745 ± 0.489) and for the PhAT probe averaged amorphous spectrum were I_380_am_ (1.194 ± 0.277) and I_1096_am_ (2.607 ± 0.277). These averaged amorphous spectra were used in the determination of %CI for each batch. The %CI values obtained for all MCC batches investigated are shown in Fig. 5.

### Partial least square regression models

A PLS model was built as an alternative to the 380-method in order to avoid having to undertake milling and spectra deconvolution. Agarwal et al. also used a PLS model to determine %CI (Agarwal et al. 2010). However, the present study involved a greater number of MCC batches and reflects the variability across commercial batches, while the study published by Agarwal et al. included several blends including commercial batches as received and their reference milled sample, at different mass fractions.

**Fig. 6:**
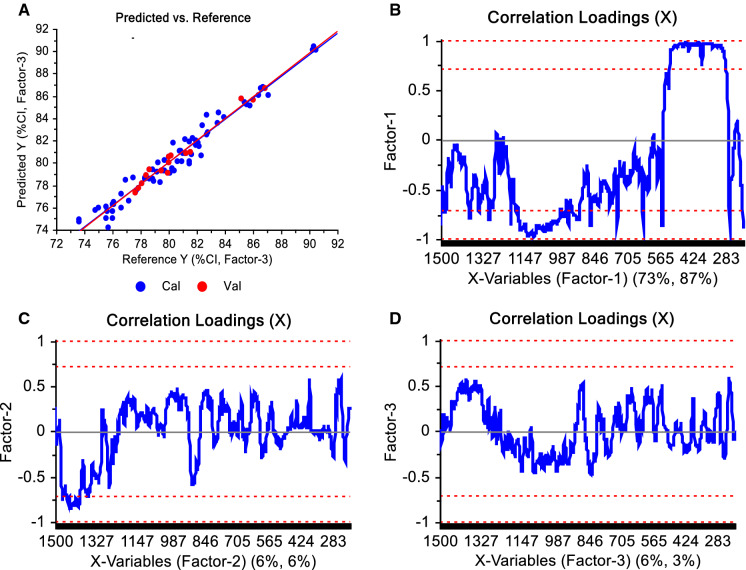
**a** Values of crystallinity index (%CI) predicted by the Partial Least Square Regression model vs Reference values for the MR probe and considering three factors, and **b**, **c**, **d** correlation loadings of factors 1, 2, and 3, respectively, obtained from the model designed using baselined and normalized spectra. The further the correlation loading is from the zero, the stronger the Raman shift contributed to explain the variability encountered by the factor. In blue are the calibration and in red the validation sets

**Fig. 7:**
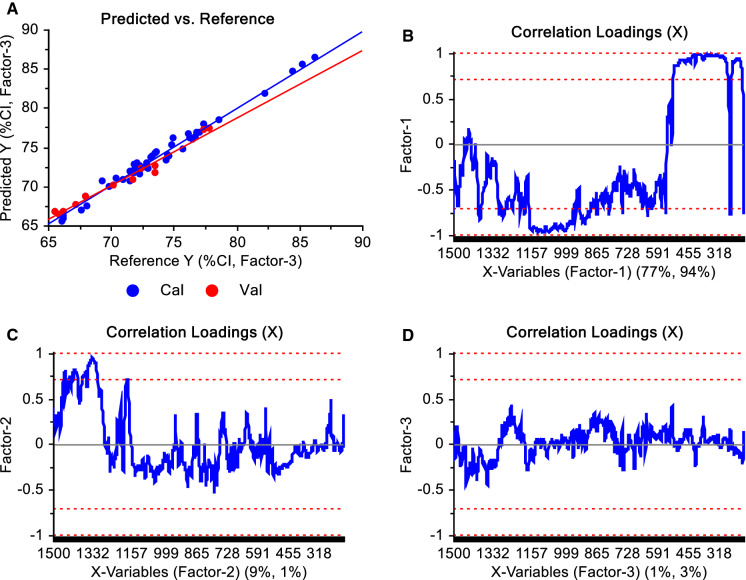
**a** Values of crystallinity index (%CI) predicted by the Partial Least Square Regression model vs Reference values for the PhAT probe and considering three factors, and **b**, **c**, **d** correlation loadings of factors 1, 2, and 3, respectively, obtained from the model designed using baselined and normalized spectra. The further the correlation loading is from the zero, the stronger the Raman shift contributed to explain the variability encountered by the factor. In blue are the calibration and in red the validation sets

Statistically significant PLS models were determined for both probes (Table 1, Figs. 6 and 7). The optimal number of factors for both models was considered to be three, which represented 97.47% of variance for the MR probe data and 97.16% of the variance for PhAT probe data. The variability captured by the first factor of both models included the Raman shifts known to be correlated to MCC crystallinity (also used to calculate MCC crystallinity by the 380-method), which was not surprising because the independent variable used to build the model was the %CI from the 380-method. This can be seen in the correlation loadings where the Raman shifts that fall within the upper or lower outer lines are the Raman shifts used by that factor to build the model (Figs. 6b–d and 7b–d).

**Table 1:** Summary statistics of the partial least square regression models

	MR probe			PhAT probe		
Number of calibration samples	67			48		
Number of validation samples	18			12		
Optimal number of factors	3			3		
N	1	2	3	1	2	3
RMSEC	1.339	0.978	0.703	1.132	1.026	0.612
RMSEP	1.346	0.597	0.460	1.212	1.1843	0.790
Explained variance (Calibration) (%)	86.97	93.05	96.40	93.84	94.94	98.20
Explained variance (validation) (%)	78.31	95.73	97.47	93.32	93.62	97.16
Bias	− 1.092	− 0.065	0.015	− 0.543	− 0.457	− 0.068

N is the Number of factors, RMSEC is the Root Mean Square Error of Calibration, RMSEP is the Root Mean Square Error of Prediction

**Fig. 8:**
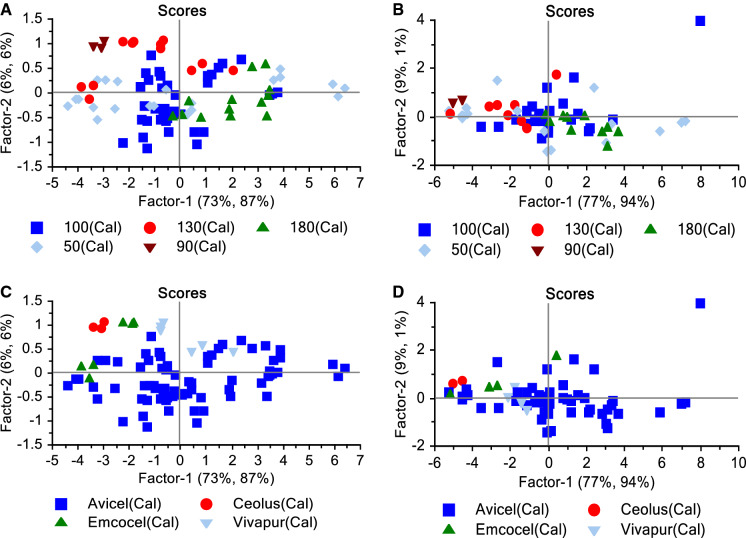
Scores plots of factors one and two highlighted by **a** and **b** average particle size in µm, and **c** and **d** manufacturer for the calibration test sets of the MR and PhAT probes, respectively

The scores plot of the PLS models was used to investigate spectral differences between batches of different average particle size and grades. However, the model was not able to separate the batches by average particle size nor grade, i.e. the PLS model was not able to identify patterns in the Raman spectra to cluster the batches in groups of the same average particle size nor groups of the same grade (Fig. 8).

## Discussion

It is challenging to determine the properties of microcrystalline cellulose compared to other materials. MCC shows great variability due to its natural source, i.e. wood pulp, and the different processes from which the commercial grades are produced, especially different drying processes. In this study the application of two models to determine the crystallinity index of MCC commercial batches was investigated. Both models showed predictive power.

The crystallinity of MCC was firstly determined using the 380-method proposed by Agarwal et al. (Agarwal et al. 2010). A calibration curve was developed using %CI values of a calibration set of blends determined by PXRD and used to correct the model for each instrument employed in this study (MR and PhAT probes, Kaiser Optical Systems Inc., USA). This modification was previously performed for a different instrument (Foster et al. 2018). Reference amorphous spectra for each batch were produced and an averaged spectrum (n = 30 batches) was determined for each probe. As a result, the production of a reference amorphous material and spectral subtraction for new batches are no longer required for future analysis. The time for analysis was shortened and milling and PXRD analysis steps (required to confirm that the sample is indeed amorphous) can be eliminated from the analytical procedure.

Crystallinity of MCC was also determined by partial least squares regression models. The crystallinity values used in this regression analysis were those determined using the established 380-method. The ability of the models to predict crystallinity from Raman spectra were 97.47% for MR and 97.16% for PhAT probes and the predicted values for the test set showed a small prediction error (RSMEP equal to 0.4596 and 0.7900 for MR and PhAT probe, respectively). The correlation loadings showed that the main wavenumbers used to build the model were 380, 437, 458, 521, 1,096, 1,120, 1,330, 1,340, 1,380, and 1,475 cm^−1^. Those wavenumbers were correlated to a change in cellulose crystallinity due to contributions from OH rocking and bending, CH and CH_2_ bending, CO and COC stretching, CH wagging, and anhydroglucose ring stretching, twisting, and torsion modes (Agarwal et al. 2010). The models built may be used to rapidly determine crystallinity for future MCC batches without the necessity to produce an amorphous reference spectrum. A PLS model had been previously used to predict crystallinity (Agarwal 2019; Agarwal et al. 2010), however, the model was built with only ten samples (a control, 4 mixtures, 3 samples milled during different times and 2 commercial MCC grades). The present study investigated thirty MCC commercial batches including ones with different particle sizes (from 50 to 180 µm average particle size) and MCC grades. Thus, the present study confirmed the finding of the previous study reported by Agarwal et al. and enriched the validation of the models by investigating the variability encountered in commercial grades of MCC.

In this study Raman probes that can scan large surface areas and give an averaged spectrum in a short time were used (1 min for MR probe and 15 s for PhAT probe). Previous studies used Raman spectra of microscopic surface areas (Agarwal 2019; Agarwal et al. 2010; Foster et al. 2018). The PhAT probe used in the present study acquires spectra from an area of 12.57 mm^2^ and the MR probe from an area of 7.85 × 10^–3^ mm^2^. A PhAT probe also has a depth of analysis of approximately 2 mm. Thus, less replicates are necessary to achieve a representative sample when a PhAT probe is used. As a result, Raman spectrum containing more averaged information of the overall semi-crystalline structure of MCC samples is obtained. Comparing the PhAT and the MR probes, the larger coverage area of the PhAT probe resulted in better fits for both the 380-method and PLS models. This was quantified by Pearson’s coefficients obtained from the calibration of the 380-method (Fig. 7, Pearson’s-r of 0.969 and 0.895 for PhAT and MR probes, respectively) and the PLS model (correlation of 0.982 and 0.964 for PhAT and MR probes, respectively). The variability observed between duplicate values determined using the PhAT probe was greater than when using the MR probe, for a number of the commercial samples. A possible explanation may be due to spectra being acquired on the same surface of the pellet (top surface) for the MR probe, while for the PhAT probe one spectrum was acquired on the top surface and another was acquired on the bottom surface of the pellet. The difference in %CI due to the differences in the positions from which the Raman spectra were may be due to the characteristic of uniaxial compression, which does not hold a homogeneous stress distribution in the interior of the pellet (Takeuchi et al. 2004).

It is important to also emphasis the limitations of quantifying the %CI of cellulose materials, including MCC, using the Raman spectroscopy. Key limitations include the lack of 100% crystalline or amorphous cellulose standards or references and the need to adjust model parameters for each instrumental set-up by calibration against diffraction data. Therefore, the %CI determined by Raman analysis is dependent on the diffraction analysis methodology. In this study, a simplistic approach was undertaken which mimicked that of Agarwal et al. (2010). The %CI of calibration set samples were determined using the Segal peak height method and theoretical %CI values were determined based on the %CI of a single MCC batch. It is proposed that an improvement to this approach should include a wider calibration set of samples in the calibration study to improve model robustness. It is also important recognize the limitations of the Segal peak height method employed. Driemeier and Calligaris (2011) highlighted that peak area is more representative of the crystalline fraction and French Santiago Cintrón (2013) demonstrated that the use of the Segal method to determine MCC %CI is influenced by sample crystallite size. The respective strengths and limitations of more commonly diffraction method discussed by French (2020), as are standards for conducting crystallographic work to study cellulose crystallinity (French 2020). Advancement from the Segal peak height method during calibration, towards other methods such as decomposition or deconvolution of peak area during calibration (Lanson 1997; Park et al. 2010; Ahvenainen et al. 2016; Yao et al. 2020), and Rietveld refinement based methods (Madsen et al. 2011; Ling et al. 2019) could further improve the models proposed in this study.

A R Shiny web application (McCrystal–crystallinity research^©^), was designed (*i*) to perform baseline correction and SNV normalization, (*ii*) to predict MCC crystallinity using the 380-method for the MR and the PhAT probes, (*iii*) to predict MCC crystallinity from PLS models, and (*iv*) to perform PCA analysis from Raman spectra within the range of 1500–250 cm^−1^. This application was developed based on the knowledge generated in this study. Thus, baseline correction, SVN normalization, and the PCA model can be applied to Raman spectra of microcrystalline cellulose obtained using different Raman instruments. However, the prediction of the crystallinity index can only be undertaken if a MR or a PhAT probe was used to acquire the Raman spectra. Even if those instruments are used, results should be evaluated with caution since different units of a same instrument design might require instrument-specific correction of the model.

## Conclusions

Crystallinity index was determined for 30 commercial batches of microcrystalline cellulose using two different models, i.e. 380-method and PLS regression. Both models showed adequate predictive power. However, the development of the PLS model takes substantially less time for analysis because it eliminates the need for milling and deconvolution of the spectra of the milled sample into amorphous and crystalline contributions before the actual %CI determination. For these models a general reference amorphous Raman spectrum was proposed for each instrument. Spectral comparison and principal component analysis showed that values of crystallinity index were relative to the instrument used to acquire the Raman spectra. Also, larger laser spot sizes give more reproducible and representative information on the overall crystallinity of the sample. The crystallinity index values obtained with either model depend on the XRD methodology and calibration sample set employed during calibration. The methodology presented can be further advanced by employing a broader sample set and more advanced XRD methodologies to measure microcrystalline cellulose crystallinity during calibration of the model. A web application (McCrystal–crystallinity research^©^) was developed which facilitates the use of the predictive models developed in this study to measure MCC crystallinity.

## Supplementary Information

Below is the link to the electronic supplementary material.

Supplementary file1 (DOCX 245 KB)

Supplementary file2 (DOCX 28 KB)
