# Supplementary material for: Investigating microcrystalline cellulose crystallinity using Raman spectroscopy
Source: Cellulose (Lond). 2021 Jul 27;28(14):8971–85. doi: 10.1007/s10570-021-04093-1 (PMC8550365; doi:10.1007/s10570-021-04093-1)
Supplement: Supplementary file 1 — Supplementary file1 (DOCX 245 KB) [file 10570_2021_4093_MOESM1_ESM.docx]

Table S1. Brand, Grade, average particle size and % CI of the 30 batches of microcrystalline cellulose investigated determined using PhAT and MR probes

*ND refers to not determined. The replicate crystallinity values for batch #26 could not be determined due to an instrumental error during measurement.

| Batch | Brand | Grade | Average particle size (μm) | % CI (PhAT) | % CI (PhAT) | % CI (MR) | % CI (MR) | % CI (MR) |
| --- | --- | --- | --- | --- | --- | --- | --- | --- |
|  |  |  |  | Rep. 1 | Rep. 2 | Re 1 | Rep. 2 | Rep. 3 |
| 1 | Ceolus ^TM^ | PH102 | 90 | 66.79 | 65.60 | 78.08 | 77.70 | 77.35 |
| 2 | Vivapur® | 102 | 130 | 72.24 | 71.76 | 81.05 | 80.94 | 80.54 |
| 3 | Emcocel® | 90M | 130 | 70.23 | 68.01 | 79.30 | 79.41 | 78.91 |
| 4 | Avicel® | PH102 | 100 | 72.10 | 86.19 | 78.63 | 78.32 | 78.57 |
| 5 | Avicel® | PH102 | 100 | 72.49 | 71.80 | 81.54 | 80.85 | 81.54 |
| 6 | Avicel® | PH101 | 50 | 67.75 | 65.85 | 75.98 | 75.61 | 76.59 |
| 7 | Avicel® | PH102 | 100 | 76.31 | 73.63 | 81.98 | 83.60 | 82.53 |
| 8 | Avicel® | PH102 | 100 | 73.78 | 71.90 | 79.28 | 79.32 | 80.14 |
| 9 | Avicel® | PH102 | 100 | 75.30 | 71.61 | 78.62 | 79.25 | 80.20 |
| 10 | Avicel® | PH101 | 50 | 67.00 | 65.99 | 75.04 | 76.33 | 75.66 |
| 11 | Avicel® | PH102 | 100 | 72.93 | 72.11 | 78.53 | 79.32 | 80.12 |
| 12 | Avicel® | PH102 | 100 | 71.82 | 73.52 | 79.09 | 80.72 | 80.01 |
| 13 | Avicel® | PH102 | 100 | 78.46 | 76.45 | 85.51 | 85.23 | 86.05 |
| 14 | Avicel® | PH102 | 100 | 70.81 | 69.85 | 78.32 | 78.56 | 76.26 |
| 15 | Avicel® | PH102 | 100 | 76.71 | 74.51 | 81.95 | 80.87 | 81.80 |
| 16 | Avicel® | PH102 | 100 | 72.53 | 72.26 | 77.56 | 79.41 | 79.05 |
| 17 | Avicel® | PH102 | 100 | 67.51 | 66.25 | 74.93 | 76.01 | 77.17 |
| 18 | Avicel® | PH101 | 50 | 76.80 | 69.31 | 79.96 | 78.51 | 78.57 |
| 19 | Avicel® | PH101 | 50 | 85.61 | 72.32 | 90.06 | 90.44 | 90.14 |
| 20 | Avicel® | PH101 | 50 | 71.89 | 72.03 | *ND | *ND | *ND |
| 21 | Avicel® | PH101 | 50 | 81.76 | 84.48 | 80.81 | 80.25 | 81.07 |
| 22 | Avicel® | PH101 | 50 | 65.52 | 71.48 | 74.74 | 75.13 | 74.23 |
| 23 | Avicel® | PH101 | 50 | 77.90 | 76.32 | 86.10 | 86.82 | 86.70 |
| 24 | Avicel® | PH200 | 180 | 77.34 | 77.38 | 85.66 | 85.73 | 86.68 |
| 25 | Avicel® | PH200 | 180 | 76.83 | 74.90 | 85.13 | 84.34 | 84.11 |
| 26 | Avicel® | PH200 | 180 | 73.39 | 71.89 | 79.57 | *ND | *ND |
| 27 | Avicel® | PH200 | 180 | 74.16 | 73.21 | 79.99 | 81.08 | 80.69 |
| 28 | Avicel® | PH200 | 180 | 74.80 | 73.28 | 82.20 | 81.79 | 82.76 |
| 29 | Vivapur® | 102 | 130 | 71.12 | 72.29 | 83.32 | 84.51 | 82.16 |
| 30 | Emcocel® | 90M | 130 | 72.36 | 66.20 | 75.82 | 76.51 | 75.99 |

Figure S1. Representation of the interpolation between the 9 anchor points defined. The baseline subtraction is the subtraction of the spectra (black) by the line interpolated between the 9 point (red), and it was performed individually for each Raman spectrum.

Figure S2. PXRD diffractogram of MCC sample (batch #7) before ball milling (black) and after ball milling (grey). Diffractograms were collected using a 0.02° 2θ step and 35 sec/step.

Figure S3. PXRD diffractogram of blends of ‘Crystalline’ MCC (batch #7) prior to ball milling and ’Amorphous’ MCC (batch #7) after ball milling.
