# Supplementary material for: Investigating microcrystalline cellulose crystallinity using Raman spectroscopy
Source: Cellulose (Lond). 2021 Jul 27;28(14):8971–85. doi: 10.1007/s10570-021-04093-1 (PMC8550365; doi:10.1007/s10570-021-04093-1)
Supplement: Supplementary file 2 — Supplementary file2 (DOCX 28 KB) [file 10570_2021_4093_MOESM2_ESM.docx]

############################################

# This program is linked to the publication:

##"Queiroz, A.L.P., Farag, F., Faisal, W., Kerins, B.M., Yadav, J., Healy, A.M., Crowley, M.E., Vucen, S. and Crean, A.M., 2021.

##Determination of crystallinity variability in commercial batches of microcrystalline cellulose using Raman spectroscopy. Cellulose."

# Description: Determination of relative crystallinity of microcrystalline cellulose from Raman spectral data

# First version: 28/10/2019

# Last revision: 08/05/2021 online version

# @author Ana Luiza Pinto Queiroz pqueiroz.ana@gmail.com

#############################################

## Check for and or install dependencies

library(shiny)

library(shinydashboard)

library(readr)

library(ggplot2)

library(prospectr)

library(RcppArmadillo)

library(pls)

library(pracma)

library(spftir)

library(spectrolab)

library(cluster)

library(basicTrendline)

options(shiny.maxRequestSize = 30*1024^2) # Increase the maximum size of files that can be uploaded

## Set user Interface

ui <- dashboardPage(skin = "black", title = "MCCrystal" , #this tittle is the tab name to bookmark

dashboardHeader(disable = TRUE

),

dashboardSidebar(

tags$br(),

radioButtons(inputId = "MR_or_PhAT",label = "Probe type:",

choices = c("PhAT probe", "MR probe")),

fileInput(inputId = "file",

label = "Choose a . CSV file containing MCC Raman spectra",

accept = c('text/csv', 'text/comma-separated-values, text/plain', ".csv")

),

helpText(p("Ensure that the Raman shifts are displayed in the first row and name of the samples in the first column."), p(" Each and every spectrum should be within the interval 1500-200 1/cm"),style = "color:white"),

width = 273

),

dashboardBody(tabsetPanel(type = "tabs",

tabPanel(title = "Crystallinity index results",

sidebarPanel(downloadLink("downloadcrystallinity", "Download Results"),

width = 3

),

mainPanel(

tableOutput("results")

)

),

tabPanel("Spectral Pre-treatment",

sidebarPanel(

numericInput(inputId = "no_replicates",label="Choose the number of replicates of each sample", value = 1, min = 1, max = 30),

strong(helpText("All samples must have the same number of replicates.")),

tags$hr(),

downloadLink("downloadSNV","Download pre-processed spectra"),

strong(helpText("Baseline and Standard Normal Variate were the pre-processing performed")),

width = 3

),

mainPanel(

plotOutput("snv_spectra"),

#downloadLink("snv_plot", "Download SNV/Baselined Plot"),

plotOutput("raw_spectra"),

#downloadLink("raw_data_plot", "Download Raw Data Plot")

)

),

tabPanel("Principal Component Analysis",

sidebarPanel(

numericInput(inputId = "PC_X", label = "Principal component in x-axis", value = 1, min = 1, max = 7),

numericInput(inputId = "PC_Y", label = "Principal component in y-axis", value = 2, min = 1, max = 7),

strong(helpText("Refer to Scree Plot to determine the optimum maximum number of components")),

br(),

br(),

br(),

br(),

br(),

br(),

br(),

br(),

br(),

br(),

br(),

br(),

numericInput(inputId = "PC_loading",label = "Loadings of component", value = 1, min = 1, max = 7),

br(),

br(),

br(),

br(),

br(),

br(),

br(),

width = 3

),

mainPanel(

fluidRow(splitLayout(cellWidths = c("50%", "50%"),

plotOutput("scoresplot"), plotOutput("scoresplot2"),

width = 850)

),

tags$br(),

fluidRow(splitLayout(cellWidths = c("70%", "40%"),

plotOutput("loadings"), plotOutput("scree"),

width = 850)

)

)

),

tabPanel("Partial Least Squares Regression",

textOutput("PLSsummary"),

verbatimTextOutput("summary_PLS"),

),

tabPanel("Raw data - headings",

textOutput("structure"),

tableOutput("read_data")

)

))

)

## Set the process

server <- function(input, output, session){

## Set up reactive objects

# Load raw data

df0 <- reactive({read.csv(input$file$datapath, header = FALSE)})

PLS_PhAT<-reactive({read.csv("www/PLS_PhAT.csv", header = TRUE)})

PLS_MR<-reactive({read.csv("www/PLS_MR.csv", header = TRUE)})

#Delete the first row and column of the raw data, i.e. the Raman_shifts and the IDs to prepare df0 for calculations

df1 <- reactive({df0()[-1,-1]})

# Extract Raman_shifts

Raman_shifts <- reactive({as.numeric(df0()[1,-1])})

# Create a vector with observations IDs, i.e. vector with labels for plots

observation_ID <- reactive({(df0()[-1,1])})

#Perform baseline correction

baselined_spec <- reactive({

# Exclude the first column (observations ID) so the data can be used in calculations

data <- df0()[,-1]

#Create a matrix of zeros where the resulting baseline spectra will be stored after the loop

baselined_spec <- matrix(0, nrow = nrow(data), ncol = ncol(data))

#Populate the first row with the wavenumbers

baselined_spec[1,] <- as.matrix(data[1,])

#perform spmbl line by line and store the result in the matrix created previously

for (row in 2:nrow(data)){

baselined_spec[row,] <- as.vector(spmbl(spectrum = as.matrix(data[c(1,row),]), lbl = c(1500, 1200, 952, 857, 743, 632, 550, 260, 200))[2,])

}

#Convert matrix to dataframe

as.data.frame(baselined_spec)

})

# Performe standardNormalVariate to baselined spectra

snv_spec <- reactive({

std <- standardNormalVariate(X=baselined_spec()[-1,])

})

#Create averaged spectra from no of replicates

mean_snv_spec <- reactive({

std <- standardNormalVariate(X = baselined_spec()[-1,])

# Average raw spectra

avgspectra = function(df, n = input$no_replicates){ #average each "no_replicates" rows

aggregate(x = df,

by = list(gl(ceiling(nrow(df)/n), n)[1:nrow(df)]),

FUN = mean)

}

average_spec1 <- avgspectra(std) #create a column with the group index

average_spec2 <- average_spec1[,-1]#delete the first column with the IDs

})

#Calculate crystallinity usign Agarwal model

crystallinity <- reactive({

location_380 <- which(sapply(baselined_spec()[1,], function(x) any(x == "380")))

location_1096 <- which(sapply(baselined_spec()[1,], function(x) any(x == "1096")))

################

#Extract the columns of 380 and 1096 using baselined spectra

#values_380<-as.data.frame(baselined_spec()[-1,location_380])

#values_1096<-as.data.frame(baselined_spec()[-1,location_1096])

################

#Extract the columns of 380 and 1096 using baselined/SNV spectra

values_380 <- as.data.frame(snv_spec()[,location_380])

values_1096 <- as.data.frame(snv_spec()[,location_1096])

#Calculate crystallinity

#Crystallinity <- as.data.frame(((values_380-value380amorphous)/(values_1096-values857amorphous)-0.0286)/0.0065)

if(input$MR_or_PhAT == "PhAT probe")

cristallinity <- as.data.frame(((values_380-(1.1936))/(values_1096-(-2.6071))+0.1291)/0.0048)

else

cristallinity <- as.data.frame(((values_380-(0.6271))/(values_1096-(-2.745))+0.1339)/0.0054)

#Convert matrix to dataframe

crystallinity <- data.frame(observation_ID(),cristallinity)

crystallinity

})

# Recover the data from the package CRytallinitY

xVariables <- reactive({

if(input$MR_or_PhAT == "PhAT probe")

xVariables<-as.matrix(PLS_PhAT()[-1,2:1302])

else

xVariables<-as.matrix(PLS_MR()[-1,2:1302])

xVariables

})

yVariable <- reactive({

if(input$MR_or_PhAT == "PhAT probe")

yVariable<-as.matrix(PLS_PhAT()[-1,1])

else

yVariable<-as.matrix(PLS_MR()[-1,1])

yVariable

})

#perform PLS regression

CRYSpls <- reactive({

CRYSpls<-pls::plsr(yVariable()~xVariables(), ncomp=3,validation = "CV")

CRYSpls

})

#prediction

CI_pls <- reactive({

xVariables<-as.matrix(snv_spec())

CI_pls <-as.matrix(predict(CRYSpls(), newdata=xVariables, ncomp = 3))

CI_pls

})

#Perform PCA analysis

pca <- reactive({stats::prcomp(snv_spec(), scale=TRUE,center = TRUE)})

#create a data frame with the scores of the 2 components that the user chose

scores <- reactive({data.frame(X = pca()$x[,input$PC_X], Y=pca()$x[,input$PC_Y])})

#create a data frame with scores of PC1

scores_PC1 <- reactive({data.frame(X = pca()$x[,1])})

# Determine how much variation in the original data each principal component accounts for, i.e. the explained variance of each PC

#Express pca.var in percentage values

pca.var.per <- reactive({round((pca()$sdev^2)/sum(pca()$sdev^2)*100, 1)})

#Plot scores of PC1 vs batch number

no_observation <- reactive({as.data.frame(1:nrow(df1()))})

#create a data frame with the scores of the 2 components that the user chose

#scores_line_series <- reactive({data.frame(X=no_observation(), Y=pca()$x[,1])})

# Display crystallinity

results <- reactive({

results <- data.frame(crystallinity(),CI_pls(),scores())

colnames(results) <- c("Sample ID","380-Method Crystallinity Index (%)","PLS Crystallinity Index (%)","Scores PC-1","Scores PC-2")

results

})

## Build outputs

# Plot Raw Spectra

output$raw_spectra <- renderPlot({

req(input$file)

matplot(Raman_shifts(),t(df1()),type="l", xlab = "Raman shift (1/cm)", ylab = "Intensity",main="Raw Spectra")

})

# Plot SNV Spectra

output$snv_spectra <- renderPlot({

req(input$file)

matplot(Raman_shifts(),t(mean_snv_spec()),type="l",xlab = "Raman shift (1/cm)", ylab = "Intensity",main="SNV and Baselined Spectra")

})

# Create a table of baselined/SNV spectra to be downloaded

download_SNV <- reactive({

download_SNV <- data.frame(observation_ID(),snv_spec())

colnames(download_SNV) <- c("Sample ID",Raman_shifts())

download_SNV

})

# Downloadable baselined/SNV spectra

output$downloadSNV <- downloadHandler(

filename = function() {paste("baselined-SNV-", Sys.Date(), ".csv", sep="")},

content = function(file) {write.csv(download_SNV(), file)}

)

# Create a table of results

output$results <- renderTable({req(input$file)

results()

})

# Download results table

output$downloadcrystallinity <- downloadHandler(

filename = function() {paste("Results-", Sys.Date(), ".csv", sep="")},

content = function(file){

write.csv(results(), file)

}

)

#Plot scores

output$scoresplot <- renderPlot({req(input$file)

ggplot(scores(), aes(scores()$X, scores()$Y, label=observation_ID())) +

geom_hline(yintercept=0, linetype="dashed", color = "black")+

geom_vline(xintercept = 0, linetype="dashed", color = "black")+

geom_text() +

xlab(paste("PC",input$PC_X," - ", pca.var.per()[input$PC_X], "%", sep="")) +

ylab(paste("PC",input$PC_Y," - ", pca.var.per()[input$PC_Y], "%", sep="")) +

theme_bw()+

labs(title = "Scores plot")+

theme(plot.title = element_text(face="bold",hjust = 0.5, size = 16),

axis.title.x = element_text(size=14),

axis.title.y = element_text(size=14))# Center title position and size

})

output$scoresplot2 <- renderPlot({req(input$file)

matplot(no_observation(),pca()$x[,1], xlab = "Sample number", ylab = "Scores of PC1", type = "l")

abline(h = 0, col = "black", lty=2)

})

#Plot Scree

output$scree <- renderPlot({req(input$file)

first_four <- pca.var.per()[1:4]

bp <- barplot(

first_four,

xlab = "Principal Component",

ylab = "Explained Variance (%)",

ylim = c(0,100),

names.arg = c("PC1","PC2","PC3","PC4"),

border = "black",

width = 1)

#Add values to the bars

text(bp, 0, round(first_four, 1),cex=1,pos=3)

})

#Plot loadings

output$loadings <- renderPlot({req(input$file)

matplot(Raman_shifts(),pca()$rotation[,input$PC_loading], xlab = "Raman shift (1/cm)", ylab = paste("Correlation Loadings of PC-",input$PC_loading),type="l")

abline(h = 0, col = "black", lty=2)

})

#Give information on the PLS model used

output$PLSsummary <- renderText({req(input$file)

dimension <- dim.data.frame(df0())

print(paste("Summary statistics of the PLS model used to predict the crystallinity index values. This model was built from Raman spectra colleted using a", input$MR_or_PhAT))

})

#PLS summary

output$summary_PLS <- renderPrint({

req(input$file)

s <- summary(CRYSpls())

})

#Display raw data

output$read_data <- renderTable({

req(input$file)

data1 <- read.csv(input$file$datapath, header = TRUE)

head(data1)

})

#Count rows and columns and print the data dimension

output$structure <- renderText({req(input$file)

dimension <- dim.data.frame(df0())

print(paste("Raw data contains", dimension[1], "observations and ", dimension[2], "variables."))

})

}

shinyApp(ui, server)
